# Pre-existing influenza antibodies, younger age, and increased CD4 T_E+EM_ predict influenza vaccination responses in transplant recipients

**DOI:** 10.1016/j.humimm.2026.111721

**Published:** 2026-03-10

**Authors:** Christiane Rollenhagen, Gomathy Parvathinathan, Margaret R. Stedman, Geetha Chalasani, Kelly A. Birdwell, M.Lee Sanders, Mohan Ramkumar, Lauren E. Higdon, Naiqing Ye, Jefferson J.S. Santos, Scott E. Hensley, Jonathan S. Maltzman

**Affiliations:** a Geriatric Research Education and Clinical Center, Veterans Administration Palo Alto Health Care System, Palo Alto, CA, USA; b Department of Medicine, Division of Nephrology, Stanford University, Palo Alto, CA, USA; c Department of Medicine, Pittsburgh VA Medical Center, Pittsburgh, PA, USA; d Department of Medicine, Tennessee VA Medical Center, Nashville, TN, USA; e Department of Medicine, Iowa VA Medical Center, Iowa City, IA, USA; f Department of Microbiology, Perelman School of Medicine, University of Pennsylvania, Philadelphia, PA, USA

**Keywords:** Vaccination, Kidney Transplant, Influenza

## Abstract

Recipients of kidney transplants require lifelong immunosuppression therapy which is associated with a reduced response to vaccinations. We conducted a longitudinal study of influenza vaccination in US Veteran kidney transplant recipients and correlated demographic factors and T cell characteristics to the immunological outcome of the vaccination. Our data suggest that a consistent history of annual vaccination during the 3 years prior is linked to an increase in influenza antibodies prior to vaccination. High influenza titers post-vaccination are associated with younger age, increased CD4 T_E+EM_ cells and pre-existing anti-influenza IgG levels but no association of CMV serostatus or immunologically aged T cells was detected. Thus, preexisting IgG antibodies, age, and CD4 T_E+EM_ cells could serve as predictors for the successful influenza vaccination in this at-risk population informing targeted interventions to improve vaccine responses, prevent infections, and reduce influenza-associated comorbidities.

## 1. Introduction

Kidney transplantation is life-extending, but recipients of kidney transplants continue to require lifelong immunosuppression therapy, which is associated with an increased risk for opportunistic infections, reactivation of latent viral infection, and a reduced response to vaccinations. Antibody production with viral neutralization capacity leading to protection from disease is a desired outcome of vaccination. Antibody levels may provide a marker for immunogenicity and are often associated with protection from disease [1,2]. Vaccine-induced antibodies may be capable of neutralizing pathogens and initiating anti-microbial innate immune responses. Recent vaccine studies also involve measurement of T cell responses, the induction of effector functions, and the activation of innate cells such as macrophages and neutrophils [3,4]. T cell correlates may be especially important in immunosuppressed individuals and may be a better marker of immunogenicity compared with antibody production in COVID-19 studies of kidney transplant recipients [5,6]. These efforts are especially valuable for immunologically aged populations, such as kidney transplant patients with compromised T cell function. These patients typically require vaccines that deliver high doses of antigen together with adjuvants.

Primary or reactivation post-transplant cytomegalovirus (CMV) results in significant morbidity and mortality [7]; CMV has been associated with allograft glomerulopathy [8], allograft rejection [9], viral disease, and other concurrent microbial infections [10]. Latent CMV significantly shapes the immune repertoire and leads to accelerated immune aging or immunosenescence in recipients of kidney and heart transplants [11,12]. These findings suggest that latent CMV has the capacity to impair vaccine responses through its association with immunosenescence. While transplantation with pharmacological immunosuppression negatively impacts the efficacy of vaccines, the role of prior CMV infection on vaccine responses remains controversial, especially in older adults [13,14].

The process of immunosenescence has effects on all aspects of the T cell compartment. Frequencies of naïve T cells decline while those of terminally differentiated memory T cells increase [15]. CD8^+^ T effector memory re-expressing RA (TEMRA) cells lack expression of the lymphoid homing receptor CCR7 and are significant producers of proinflammatory cytokines [16–18]. In memory, TEMRA and effector T cell populations, the relative percentage of T lymphocytes expressing the terminal differentiation markers CD57 and Killer cell lectin-like receptor G1 (KLRG1) is increased [11,19–21].

To better understand preexisting factors that lead to a more effective vaccine response, we conducted an observational influenza vaccination study in US Veterans who were kidney transplant recipients (KTR). We examined whether age, consistency of annual vaccinations, time post-transplantation, CMV status, and T cell population frequencies correlated with influenza vaccine responses in this at-risk population.

## 2. Material and Methods

This study was approved by the Stanford University IRB (protocol #48771). This study was performed in accordance with the Declaration of Helsinki and all participants gave written informed consent prior study inclusion.

### 2.1. Study population

Seventeen KTRs were enrolled prior to their annual influenza vaccination at the Veterans Administration Palo Alto Health Care System between 2020–2023. One enrolled transplant recipient had active CMV viremia during the study time and was excluded. Of the 16 remaining subjects, 11 were CMV seropositive and 5 were CMV seronegative prior to kidney transplantation (Table 1). All participants were male and received immunosuppressive therapy which was adjusted based on standard of care by the treating physicians (Table 1).

### 2.2. Influenza vaccines

The study period from 2020 to 2023, encompassed 3 Influenza seasons. The vaccines administered varied by year. Vaccines administered included Fluarix (used in season 2020–21), Fluad (used in season 2021–22 and 2022–23), Fluzone (used in season 2020–21) and Afluria (used in season 2022–23). All vaccines were quadrivalent, designed to protect against four different flu viruses, including two influenza A viruses and two influenza B viruses. All vaccines included the influenza strain B/Phuket/3073/2013 which was used as viral input for the Focus Reduction Neutralization Test (FRNT) assay to assess neutralizing antibodies.

### 2.3. Blood Collection and Processing

Blood samples from each participant were collected within a week prior to vaccination, as well as at days 7, 14, 28 and 182 post-vaccination. Blood was collected in vacutainer tubes with EDTA (Ethylenediaminetetraacetic acid). Peripheral Blood Mononuclear cells (PBMCs) were isolated using SepMate PBMC isolation tubes (StemCell Technologies, USA), frozen at 5 × 10^6^ cells/mL in FBS (Gemini, USA) with 10 % DMSO (Sigma Aldrich, USA) and stored in liquid N2 as previously described [22]. Blood plasma was collected from each sample by centrifugation at 1000 × g for 15 min and was and stored at − 80 °C.

### 2.4. Flow cytometric Phenotyping of PBMCs and compensation controls

PBMC were thawed, rested overnight at 37 °C, and stimulated with overlapping peptide libraries consisting of 15 amino acid peptides with 11 amino acid overlap for the lengths of the proteins for CMV immediate early-1 (IE-1) and pp65 as described [23,24]. Cells were stained as described previously [11] using antibodies as listed in Sup Table 1. Samples were acquired with the use of a BD LSR Fortessa analyzer using FACSDiva software (BD, Franklin Lakes, NJ) (Becton Dickinson, Franklin Lakes, NK) configured for 18-color analysis at the Palo Alto VA Flow Cytometry Core as described [11]. Flow cytometric data were analyzed in FlowJo version 10.7.1 (BD, Ashland, OR). Graphs were generated and statistics calculated in GraphPad Prism (San Diego, CA) and RStudio version 2023.12.1 + 402.

### 2.5. Influenza HA enzyme-linked immunosorbent assays (ELISAs)

ELISAs were carried out as previously described [25,26]. Plates were coated with phosphate-buffered saline (PBS) or recombinant HA protein from B/Phuket/3073/2013 at 2 μg/mL in DPBS at 4 °C overnight. Blocking and dilution buffer consisted of 1x PBS with 0.1 % Tween 20, 0.5 % non-fat dry milk powder, and 3 % goat serum. The next day, ELISA plates were blocked for 2 h. Plates were washed with PBS containing 0.1 % Tween 20 (PBS-T), and 50 μL of serially diluted serum samples were added to each well. After 2 h of incubation, plates were washed with PBS-T, and 50 μL of diluted horseradish peroxidase (HRP)-conjugated anti-human secondary antibody (Jackson ImmunoResearch) was added to each well. After an hour incubation, the plates were washed with PBS-T. All plates were developed by adding SureBlue TMB peroxidase substrate (SeraCare, Gaithersburg, MD) for 5 min at RT, followed by stopping the reaction with 250 mM hydrochloric acid. The absorbance was measured at 450 nm using a SpectraMax ABS Plus plate reader (Molecular Devices, San Jose, CA). Background OD values from the plates coated with PBS were subtracted from the OD values from plates coated with recombinant HA protein. Endpoint titers were calculated as the reciprocal serum dilution that generated an equivalent optical density (OD) of 0.5.

### 2.6. Influenza virus neutralization assay

Neutralizing antibodies were measured against the B/Phuket/3073/2013 influenza strain using a focus reduction neutralization test (FRNT). FRNT assays were completed as previously described [27]. Briefly, serum samples were treated with receptor-destroying enzyme (RDE) (Denka-Seiken) for two hours at 37 °C and then inactivated for 30 min at 56 °C. Then, RDE-treated serum samples were 2-fold serially diluted in a 96-well plate using serum-free MEM and mixed with ~300 FFUs of virus per well. After 1 h incubation at room temperature, virus–serum mixtures were transferred to confluent monolayers of MDCK-SIAT1 cells. After 1 h incubation at 37 °C in 5 % CO2, cells were washed with serum-free MEM and overlaid with MEM supplemented with 5 mM HEPES buffer, 50 μg/mL gentamycin-sulfate, and 1.25 % Avicel. Plates were incubated for 18 h at 37 °C in 5 % CO2 and then fixed with 4 % paraformaldehyde for 45 min at 4 °C. Fixed cells were permeabilized with 0.5 % Triton X-100 for 7 min and then blocked for 45 min with 5 % milk in PBS. Foci were stained for 1 h with a mouse anti-influenza A nucleoprotein antibody (clone IC5–1B7) followed by 1 h incubation with a peroxidase-conjugated rat anti-mouse kappa antibody diluted in blocking buffer. Plates were washed 5 times with distilled water after blocking, primary, and secondary steps. TrueBlue TMB substrate (SeraCare) was added to plates and incubated in the dark for 30 min for foci development. Substrate was flicked out of wells, and plates were thoroughly dried before foci visualization and quantification on an ELISpot reader. Serum antibody titers were recorded as the greatest dilution that neutralized ≥ 90 % of the virus, also referred to as FRNT_90_ titers.

### 2.7. Statistical analysis

We performed a descriptive comparison of subject demographics and immunogenicity at baseline by CMV status. Baseline blood draws were conducted a week or less before vaccination and were defined as day 0. We described categorical variables with frequencies and percents, and continuous variables by median [IQR]. We performed a Mann-Whitney non-parametric test to compare medians between groups. We used Spearman’s Rank or Pearson’s correlation (depending on distributional assumptions) to measure the correlation between two variables. Heatmaps were used to display correlations graphically.

We used Generalized Estimating Equations (GEE) to examine the effect of a vaccine on the primary outcome of FRNT90 titers and IgG, adjusting for the correlation in repeated measures from the same participant. In an initial power analysis for CMV we assumed a GEE model with 11 patients with 5 measures in the CMV + group and 5 patients with 5 measures in the CMV− group, having a standard deviation of 255 for the IgG outcome and a standard deviation of 12.5 for the FRNT90 continuous outcome. We assumed the correlation measures within the same patient to be 0.66. For 80 % power we could detect a mean difference of 328.8 for the IgG outcome and 16.1 for the FRNT90 outcome. In the final analyses, IgG was modeled as a continuous outcome with a log transformation. FRNT90 titer was categorized as greater than 40 or less than or equal to 40. Age, CMV status, CD4 T_E+EM_, %CD57 of CD8, %CD57 of CD4, and previous vaccination status were explored as additional covariates in the model. We additionally reconfigured the model with the outcomes based on the change in FRNT90 titer or (day0 IgG from baseline). The change was then categorized as no difference or greater than zero. The analysis was done using R version 4.4.1 (R Core Team, Vienna, Austria) within RStudio (version 2023.12.1: Posit Software, Boston, MA).

## 3. Results

### 3.1. Baseline demographics

The cohort consisted of male US Veteran KTRs. The median time since transplantation was 3.5 years (Table 1). The median age was 70 years and 69 % were receiving triple drug immunosuppressive therapy with a steroid, anti-metabolite and calcineurin inhibitor. In terms of vaccination history, 50 % had been vaccinated for influenza each of the previous three years while the remaining 50 % had received influenza vaccines only 1–2 times during the past 3 years (Table 1).

### 3.2. Younger age and a higher frequency of previous vaccinations are associated with a preexisting influenza titer

We took advantage of the fact that all vaccines used during these studies included the B/Phuket/3073/2013 strain. We measured total binding antibodies reactive to the hemagglutinin (HA) of B/Phuket/3073/2013 using ELISAs and neutralizing antibodies using a Focus Reduction Neutralization Test 90 (FRNT90) test determined by the dilution of a participant’s plasma sample that provides for a 90 % reduction of virus in culture.

**Fig. 1.**
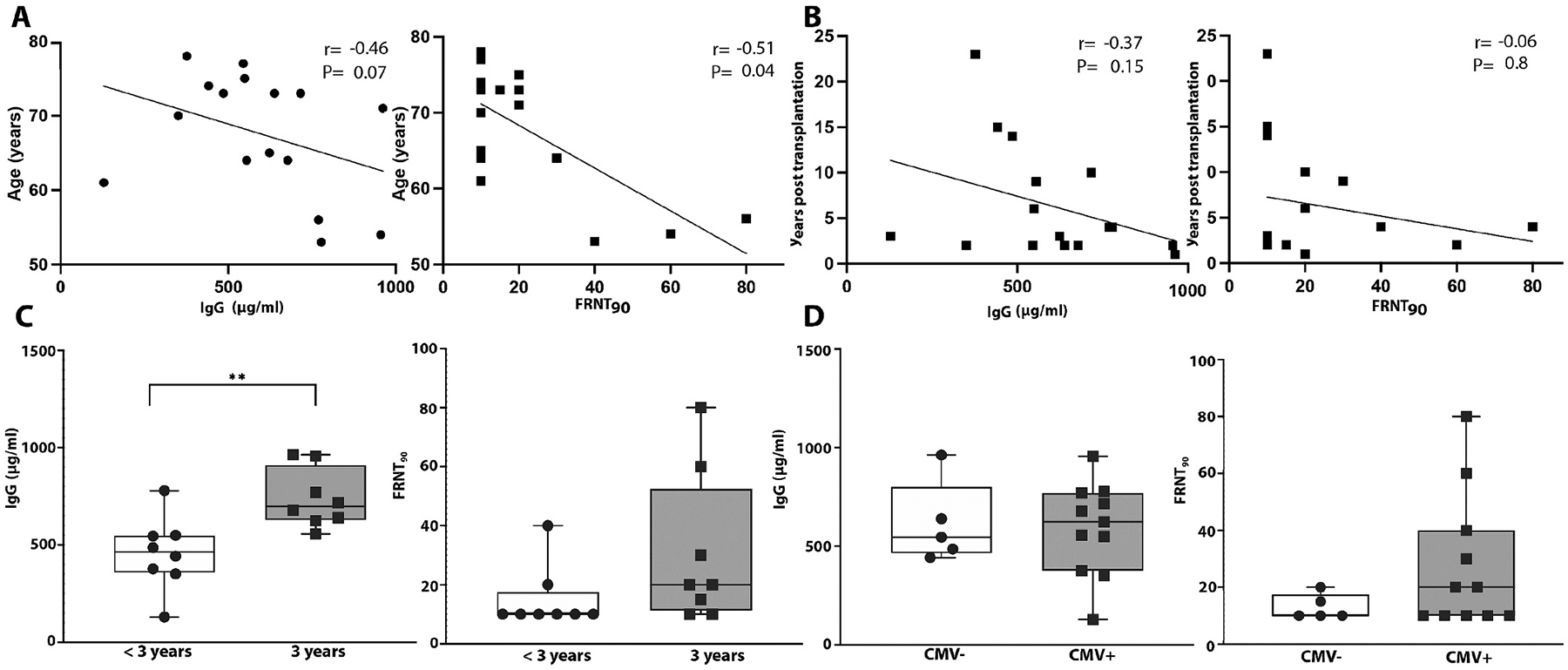
Comparison of demographics with baseline anti-influenza IgG and viral neutralization Graphs show the linear correlation between baseline IgG antibody or FRNT90 with (A) the participant’s age and (B) years’ post-transplantation. Spearman rank correlation r and p-values are shown as insets within each plot. Box plots of baseline IgG and FRNT90 stratified by (C) immunization history (clear = <3 immunizations over 3 years, gray = 3 immunizations over 3 years) and (D) CMV serostatus (clear = CMV− and grey = CMV +). Box plots depict the median and interquartile range. p-values are based on the Mann-Whitney test. *= p < 0.05, **= p < 0.005. n = 16.

To identify the characteristics associated with previous influenza vaccination we compared subject demographic information with their anti-influenza antibody concentrations prior to vaccination (Fig. 1). Age was negatively correlated with both plasma anti-B/Phuket IgG levels and FRNT90 titer (Fig. 1A). We also observed a weak negative correlation between the number of years post-transplantation and both preexisting IgG and FRNT90 (Fig. 1B). Subjects vaccinated continuously in the previous 3 years had a significantly higher level of pre-existing antiinfluenza IgG and a trend towards increased FRNT90 titer when compared to the group with fewer influenza vaccinations during that time (Fig. 1C). In contrast, there was no difference in levels of preexisting anti-B/Phuket IgG and a trend toward increased neutralization when comparing subjects based on a history of cytomegalovirus (CMV) as determined by CMV serological status (CMV^+^ versus CMV^−^) (Fig. 1D and Table 2).

### 3.3. CD4 TE + EM cells but not immunological age is positively correlated with pre-vaccination anti-influenza IgG

We next assessed whether the degree of immunological aging could be associated with pre-existing influenza antibodies and their ability for viral inhibition. We used the expression of markers CD57 and KLRG1 as surrogates for aged and terminally differentiated T cells. The percentages were determined by flow cytometry (FACS) after gating for CD4 and CD8 T cells (Supplementary Fig. S1).

**Fig. 2.**
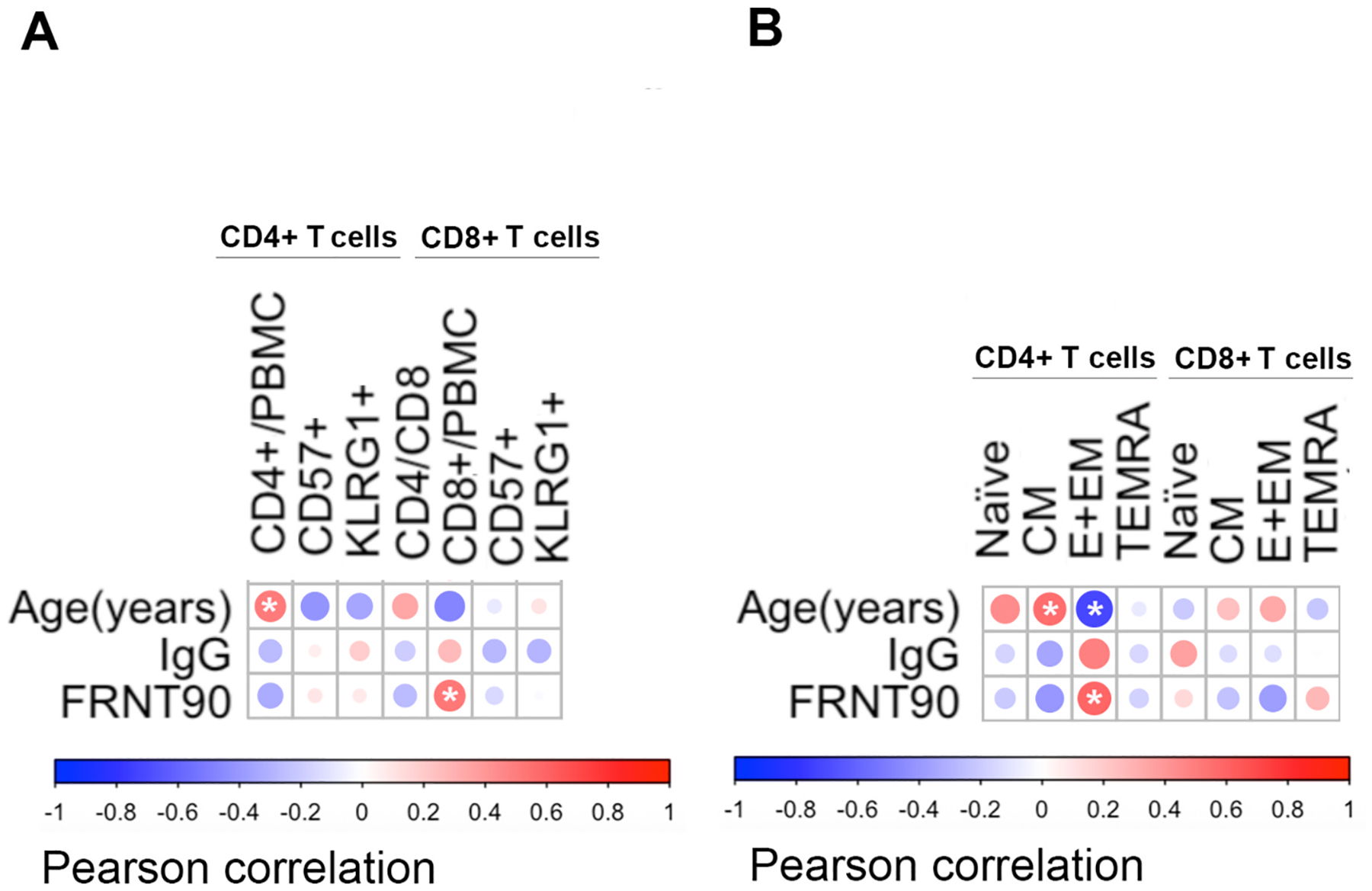
Correlations between Age, IgG and FRNT90 with T cell subsets Heatmaps of linear correlations between age, pre-existing IgG and FRNT90 and T cell populations. (A) Percent of CD4 + and CD8 + T cells and indicators or immunosenescence including CD4/CD8 ratio and expression of CD57 or KLRG1. (B) Subpopulations of CD4 + and CD8 + T cells including naïve, central memory (CM), effector and effector memory (E + EM), and effector memory T re-expresses CD45RA (TEMRA) cells. Heat maps show positive correlations in red and negative correlations in blue. Circle size and color intensity indicate the strength of the correlation. Pearson was used to determine the correlations. Asterisks indicate p < 0.05.

The participant’s age was positively correlated with the total number of CD4 T cells (Fig. 2A). Neither total CD8 + nor the expression of immune aging markers on either CD4 or CD8 T cells was significantly correlated with chronological age (Fig. 2A; row 1). When we examined the association between immune subsets with pre-existing antibody concentration and neutralization capacity, only the total number of CD8 cells was significantly correlated with FRNT90 titers (Fig. 2 A; row 3).

We next tested whether the relative frequency of T cell subpopulations correlate with levels of baseline anti-influenza IgG. We used CCR7 and CD45RA expression to define differentiation states of CD4 and CD8 T cells (Supplementary Fig. S1). Effector + Effector Memory (E + EM, CCR7-CD45RA-) CD4 T cells were significantly negatively associated while the Central Memory (CM, CCR7 + CD45RA-) were positively associated with age. Neither Naïve (CCR7 + CD45RA +) CD4, terminally differentiated effector memory re-activated T cells (TEMRA, CCR7-CD45RA +) CD4 T cells nor any CD8 T cell subpopulations were significantly correlated with age (Fig. 2B; row 1). Interestingly, CD4 T_E+EM_ cells were positively and significantly correlated with viral neutralization but not with anti-influenza IgG levels (Fig. 2B; rows 2 and 3, respectively). We further observed no significant correlations in frequency of naïve, central memory, or TEMRA T cells with anti-influenza IgG concentrations or FRNT90 titers (Fig. 2B; row 2 and 3 respectively).

### 3.4. Pre-existing anti-influenza IgG levels are associated with increased viral neutralization after vaccination

**Fig. 3.**
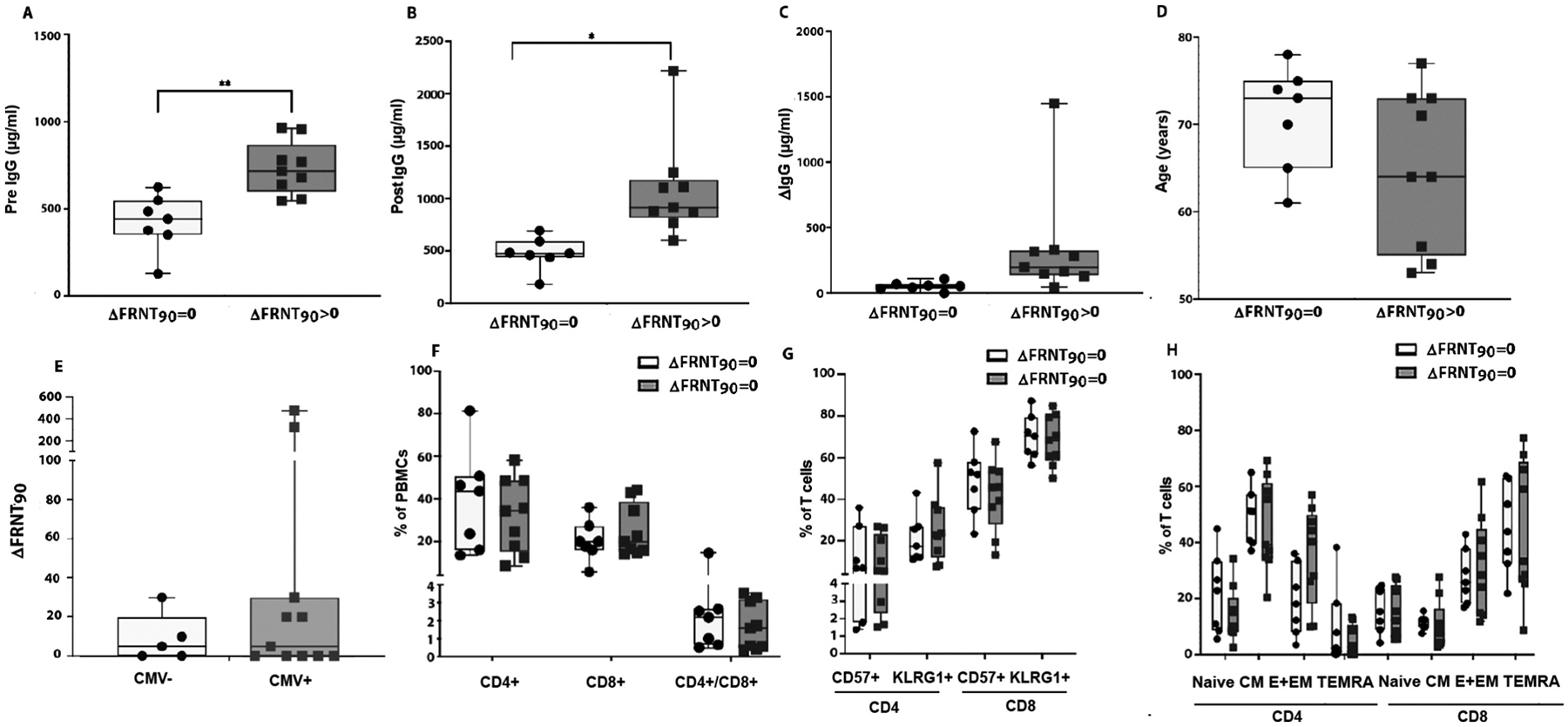
ΔFRNT90 > 0 is associated with Increased levels of IgG antibodies both before and after vaccination. Boxplots show the distribution of IgG antibody stratified by ΔFRNT90 > 0. IgG antibodies (A) before vaccination, (B) after vaccination, (C) the difference induced by vaccination, (D) participant’s age, (E) pre-transplant CMV serostatus, (F) percentages of CD4 + and CD8 + T cells and their ratio, (G) immunologically aged subsets and (H) differentiation subsets. ΔFRNT90 is calculated as maximal FRNT90 post-vaccine – FRNT90 at baseline. Open boxes are ΔFRNT90 > 0 and shaded boxes are FRNT90 = 0. N = naïve, CM = central memory, E + EM = Effector and Effector Memory, and TEMRA = T effector memory re-expressing RA cells. Boxplots show individual data points, with median and interquartile range. p-values are based on the Mann-Whitney test *= p < 0.05, **p < 0.005. n = 16.

We next assessed which immunological factors are predictive of increased vaccine induced serological changes. We collected plasma from 7, 14, 28, and 182 days after vaccination and evaluated each time point for anti-B/Phuket IgG levels and neutralization capacity. The maximum FRNT90 titer (mFRNT90) was determined for each participant. We also calculated the difference between mFRNT90 and the baseline FRNT90 titer (ΔFRNT90). We defined vaccine responders as those with a ΔFRNT90 greater than zero and non-responders with a ΔFRNT90 equal to zero. The median ΔFRNT90 in our cohort was 7.5 (Table 2). Principal Component Analysis (PCA) confirmed a separation between responders and non-responders (Supplementary Fig. S2). We next assessed whether responders and non-responders have different levels of anti-influenza IgG antibodies before and after vaccination. Non-responders had statistically lower IgG antibodies both before and after vaccination when compared with responders (Fig. 3A–B). In addition, responders had a trend toward a greater increase in anti-B/Phuket IgG levels in response to vaccination (Fig. 3C).

Consistent with the significant inverse correlation between age and influenza titers from previous vaccinations and infections (Fig. 1A), there was a non-statistical trend of younger chronological age for responders compared with non-responders (Fig. 3D). Neither CMV status, years post transplantation, nor vaccination frequency were statistically different between subjects who responded versus those that did not (Fig. 3E and data not shown). Similarly, the number of CD4 and CD8 T cells as well as aged T cell subpopulations based on expression of CD57, KLRG1 or TIGIT were not statistically significant different between responders and non-responders (Fig. 3F,G and Supplementary Fig. S3). Moreover, comparing the CD4 and CD8 T cell subpopulations between both groups revealed no differences in cell numbers (Fig. 3H).

### 3.5. Younger age and increased CD4 TE + EM are associated with high FRNT90 titers after vaccination

**Fig. 4.**
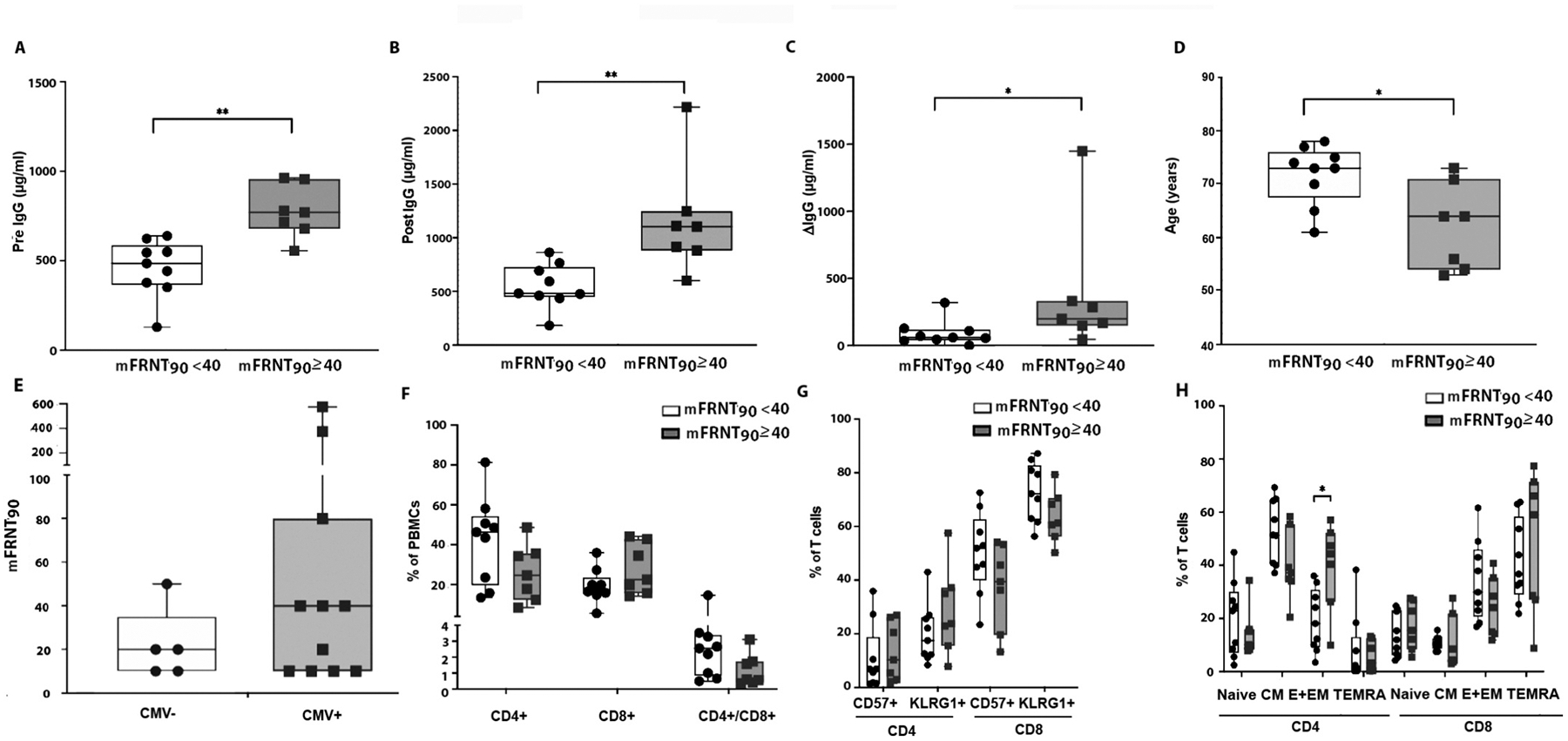
Younger Age and increased CD4 T_E+EM_ cells are positively linked to a protective titer after vaccination. Boxplots show the distribution of IgG antibody stratified by a maximal FRNT90 after vaccination. Individual plots assess IgG antibodies (A) before vaccination, (B) after vaccination, (C) the difference induced by vaccination, (D) participant’s age, (E) pre-transplant CMV serostatus, (F) percentages of CD4 + and CD8 + T cells and their ratio, (G) immunologically aged subsets and (H) differentiation subsets. Open boxes are FRNT90 < 40 and shaded boxes are FRNT90 ≥ 40. N = naïve, CM = central memory, E + EM = Effector and Effector Memory, and TEMRA = T effector memory re-expressing RA cells. Boxplots show individual data points, with median and interquartile range. Box plot graphs include individual data points, medians, and interquartile ranges. P-values are computed by Mann-Whitney test *= p < 0.05, ** =p < 0.005. n = 16.

Next, we wanted to assess which demographic and immunological factors are predictive of higher levels of viral neutralization. Seventy percent of subjects had an increase in FRNT90 titer over baseline following vaccination; 28 % achieved their mFRNT90 by day 7 or day 14 and 42 % on day 28. The remaining subjects had no increase in FRNT90 titer following vaccination. We grouped participants based on mFRNT90 titer of 40 or greater versus those with mFRNT90 titer of less than 40. Three (18.7 %) subjects had and retained an FRNT90 titer before and after vaccination with four additional subjects reaching an FRNT90 titer after vaccination (Table 2). This finding was associated with an overall increase of median anti-influenza IgG titers of approximately 36 % (Table 2). Pre-vaccination and post-vaccination concentrations of anti-influenza IgG were statistically higher in individuals with ≥ 40 mFRNT90 (Fig. 4A, B). The change in IgG level from vaccination was also greater in individuals with mFRNT90 ≥ 40 (Fig. 4C). The participant group with a ≥ 40 FRNT90 titer is significantly younger than those with lower FRNT90 titers (Fig. 4D).

In contrast to our expectation that previous CMV exposure would decrease the immune response, CMV seropositive subjects had a trend toward higher capacity for viral neutralization (Fig. 4E). In addition, those with an mFRNT90 ≥ 40 exhibited trends toward immunologically older T cells (decreased CD4/CD8 ratio, increased percentage of CD57 + or KLRG1 + CD8 T cells) (Fig. 4F, G). Comparing the CD4 and CD8 T cell subpopulations between both groups revealed that the relative percent of CD4 T_E+EM_ cells are significantly increased in participants with a high mFRNT90 titer. The remaining CD4 T cell as well as all CD8 T cell subpopulations do not show differences in relative cell numbers between the groups (Fig. 4H).

### 3.6. No association between CMV-serostatus and anti-influenza IgG levels was detected

**Fig. 5.**
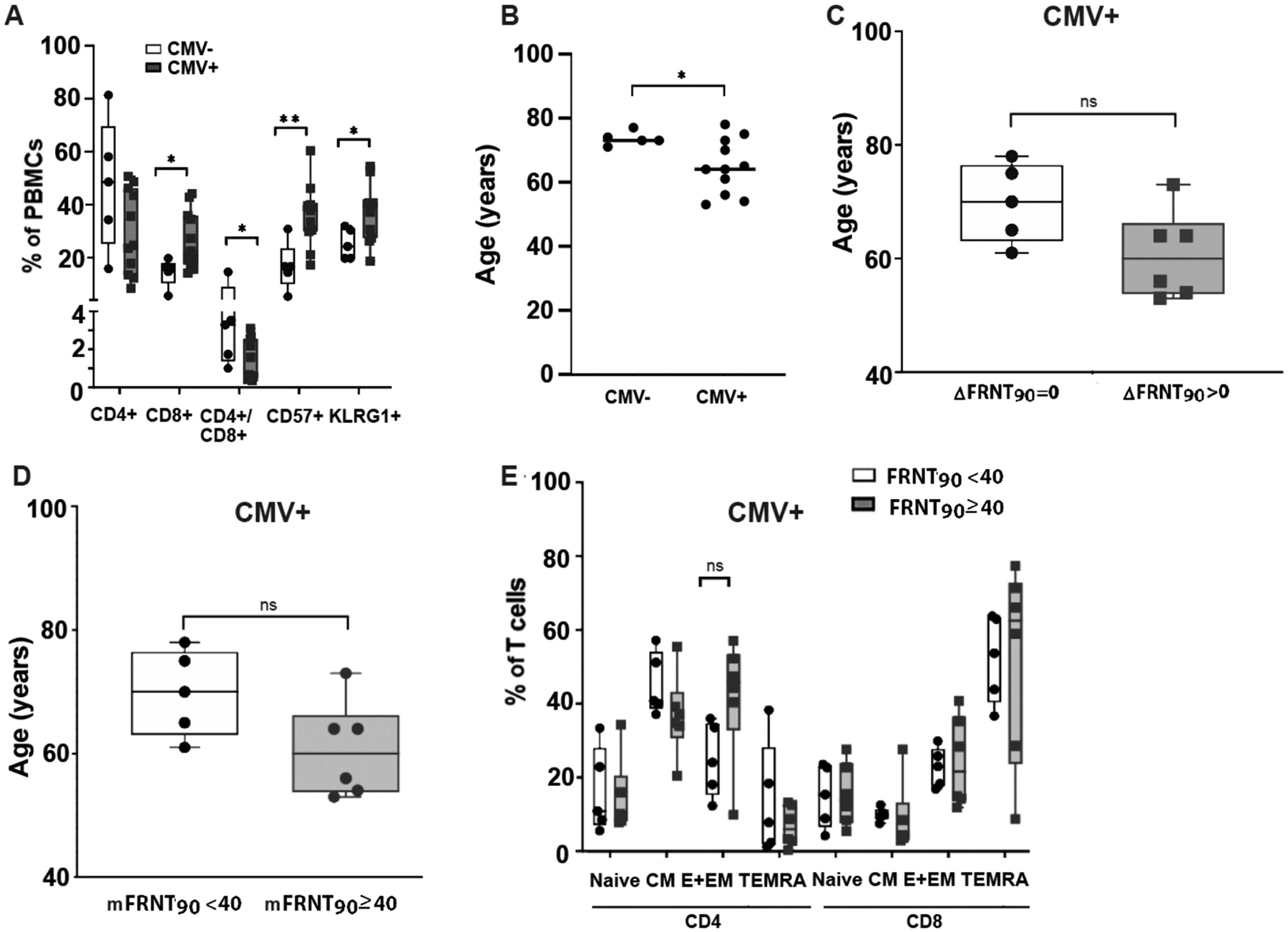
CMV + participants mimic features found in the entire population, while they have features of immune aging Box plots compare CMV + with CMV− subjects for (A) T cell subsets and (B) distribution of chronological age. Data analysis limited to CMV + subjects for age stratified by (C) ΔFRNT90, and (D) mFRNT90 titers. (E) T cell subsets from CMV + subjects comparing protective (mFRNT90 ≥ 40) with non-protective mFRNT90 < 40. Plots include individual data points, median, and interquartile range (box plots only). P-values are based on Mann-Whitney *= p < 0.05, **= p < 0.005. n = 11subjects.

Our initial hypothesis was that previous exposure to CMV as evidenced by a CMV^+^ serostatus would correlate with a diminished response to vaccination. But as shown in Figs. 3E and 4E, there was a trend toward increased neutralization capacity. Since latent CMV is associated with immune aging and significant changes in T cell characteristics, we first confirmed that CMV seropositivity correlated with markers of immune aging including a decreased ratio of CD4/CD8 T cells and increased CD57 + and KLRG1 + T cells (Fig. 5A). Further review of subject demographics demonstrated that the CMV seronegative cohort are significantly older than the seropositive group (Fig. 5B.). To address whether these changes impact vaccination outcomes and factors associated, we limited our analysis to subjects with a CMV + status. We were able to confirm that within the CMV + cohort, chronological age trended inversely with changes in viral neutralization but did not reach statistical significance (Fig. 5C, D). Limiting the analysis to CMV + participants demonstrated trends similar to the entire cohort for the CD4 T_E+EM_ subset (Fig. 5E). As the CMV seronegative subjects were all over the age of 70, we performed additional comparisons between the CMV seronegative cohort and age-matched CMV seropositive subjects from within the cohort. Despite matching for age, there was still no effect of CMV serostatus on either anti-influenza IgG or viral neutralization (Supplementary Fig. S3).

### 3.7. Statistical modeling analysis supports finding that a high post-vaccine titer correlates with younger age and increased CD4 TE + EM cells but not CMV status

**Fig. 6.**
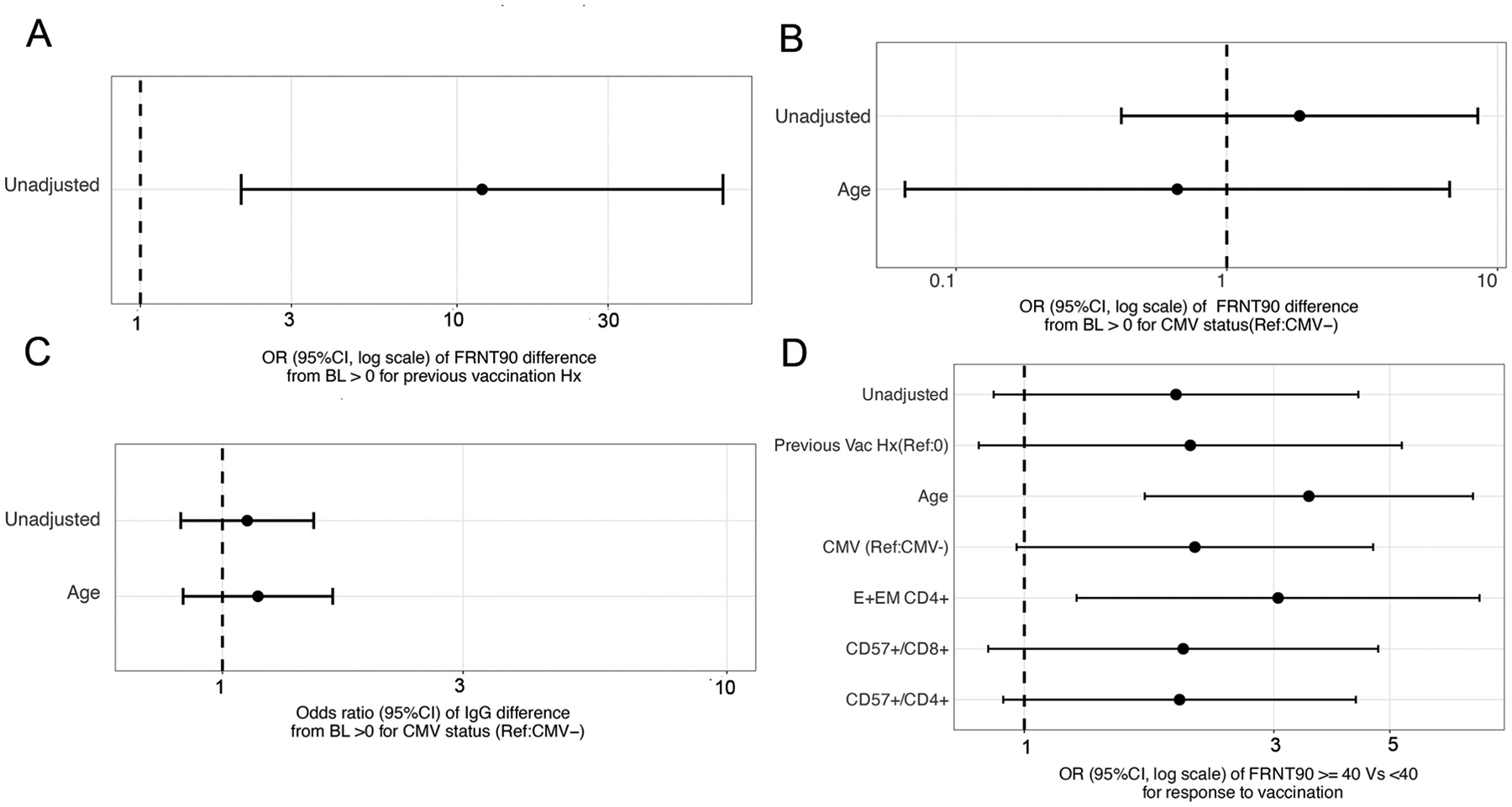
Evaluation of vaccination and longitudinal measures of IgG and FRNT90. Forest plots show unadjusted and adjusted mean differences in FRNT90 difference for previous vaccination (A), and CMV status (B), IgG difference for CMV status (C), and odds ratios of FRNT90 being greater than 40 (D) after an influenza vaccination. Generalized estimated equation (GEE) models are used to adjust for the repeated measures within participants. 95% confidence intervals are depicted around each estimate, and the dotted vertical line at 1 for odds ratios and 0 for risk differences denote no effect.

We examined the association between continuous vaccination, CMV status and longitudinal IgG and FRNT90 titer unadjusted and adjusted for confounding factors. Continuous vaccination in previous years was associated with increased viral neutralization after vaccination (Fig. 6A). The odds ratio of the difference in anti-influenza IgG and FRNT90 titers from baseline was also not significantly associated with CMV serostatus, even when adjusted for age (Fig. 6B and C).

Lastly, confirming our previous findings, we found a significant association between the vaccination event and the odds of having an FRNT90 titer ≥ 40 adjusted for age (OR = 3.5, 95 % CI (1.7, 7.2)) and for CD4 T_E+EM_ (OR = 3.1, 95 % CI (1.3,7.4)). The association between vaccination and FRNT90 consistently trended positive (OR > 2) for all models, unadjusted, and adjusted for vaccination history. However, the Ors were not significant (Fig. 6D).

## 4. Discussion

In this study, we utilized longitudinal influenza vaccine data from KTRs to determine whether CMV serostatus, chronological age, years after transplantation, continuous vaccination, immune aging, or T cell subpopulations are associated with the immunogenicity of an influenza vaccine. We found that younger age, pre-existing anti-influenza IgG and the increased amount of CD4 T_E+EM_ cells are each positively correlated with high FRNT90 titers. In contrast, CMV serostatus, previous vaccination, immune aging markers and the relative numbers of CD4 and CD8 T cells were not associated with a higher mFRNT90 titer after vaccination. Thus, age, preexisting serological immunity and increased relative frequency of CD4 T_E+EM_ cells may serve as predictors for the success of an influenza vaccine in this population.

A study in non-human primates demonstrated immune senescence in old rhesus monkeys correlated with a reduced antibody response to influenza vaccination [28]. Many laboratories confirmed this finding in human studies [29–31]. We found that in KTRs, younger chronological age is positively correlated with a higher preexisting influenza FRNT90 titer and the development of a high FRNT90 titer after vaccination. In contrast, we were unable to find an association between immune aging markers such as CD57 and KLRG1 on pre-existing or post-vaccine protective levels of viral neutralization. The presence of immunosenescent-phenotype T cells alone cannot explain the negative correlation we observed with the FRNT90 post-vaccination and increased participant’s age.

Positive CMV status has been associated with accelerated immune aging in immunocompetent and immunosuppressed humans [32]. We found a more aged immune system of CMV + subjects when compared to the CMV− in our cohort. Immune aging markers included increased CD8 + T cells and a decreased CD4+/CD8 + T cell ratio as well as increased immune cells with CD57 and KLRG1 markers. This was significant even though the participant’s average chronological age was higher in the CMV− group when compared to the CMV + group. Yet we were unable to find a difference between the CMV + and CMV− groups in regard to a pre-existing FRNT90 or the FRNT90 post-vaccination either unadjusted or adjusted for age. Together, these results suggest that features of chronological age other than aged immune cells have a greater impact on vaccine responses than immunosenescence.

The involvement of latent CMV in the antibody response to influenza vaccination is controversial. Blomberg’s group demonstrated an association between CMV-seropositivity and decreased serum response to influenza vaccination regardless of age [33]. In contrast, a *meta*-analysis of 17 studies by van den Berg et al found no association between latent CMV and the antibody response to influenza vaccination [34]. Our study was designed to address this question in older KTRs on immunosuppressive therapy. To this date the role of CMV to heterologous immune responses in this cohort is not known.

The initial antibody response to vaccination has been shown to diminish with age, and older people tend to have reduced vaccination longevity [35]. In a healthy human cohort, researchers have demonstrated the role of humoral and cell-mediated immunity for the vaccine response. They found that the risk of developing influenza after immunization was highest among the older subjects who demonstrated neither antibody nor cell-mediated responses against influenza [36]. In our study, participants with a high FRNT90 titer before vaccination were able to significantly increase anti-influenza IgG levels and viral neutralization in response to immunization, suggesting that prior exposure resulting in serological responses are an indicator of developing a strong immune response after vaccination.

Relative levels of CD4 T_E+EM_ but not immune aging markers are correlated with chronological age in our limited cohort. Aging marker expression within CD4 T_E+EM_ cells was comparable to that in the overall T cell population. These observations support previous findings that the ability to increase the level of CD4 T cells through vaccination is reduced with age leading to limiting the activation of antibody producing B cells [37].

The age-related decline in immunity reduces the efficacy of vaccinations which increases the risk of infections and mortality in older adults with transplantations. Current research is directed to improve vaccination regimes in older adults using adjuvants and interventions such as the time of day the vaccine is given or the use of booster vaccinations [38]. Our data support consistent annual vaccination in KTRs as an intervention to increase IgG antibodies and efficiency of a vaccine response.

Our study has several limitations. First and foremost, we had a small number of subjects and was thus underpowered to make statistically significant conclusions for several factors. Despite spanning 3 separate years of vaccination, we were unable to recruit additional subjects due to the COVID-19 pandemic. Importantly, our cohort was limited in the number of CMV seronegative subjects and they were all over the age of 70 confounding our ability to make firm conclusions of the effect of CMV status on vaccine response. To increase power, we opted to include all timepoints measured for each subject and adjust the analyses for the within patient correlation. Additionally, our FRNT90 data have a large range that together with the low sample size led to several trends that did not reach statistical significance including correlation between continuous vaccination and an increase in FRNT90 (data not shown). In addition, because this study recruited over a 3-year period, there were multiple vaccine preparations administered, so we only measured responses against the shared influenza vaccine component. Another important limitation is the choice of aging markers for the determination of senescent cells. While CD57 and KLRG1 are accepted markers of immune aging others such as telomere shortening and epigenetic alterations (e.g., DNA methylation) may allow for a more refined estimation of immune age require techniques which were out of the scope of this project. This study does not assess influenza-specific T-cell function, such as numbers of influenza specific tetramer positive cells or cytokine responses (e.g., IFN-γ, TNF-α, IL-2, IL-6) that drive viral clearance and could contribute to the mechanistic interpretation of CD4 TE + EM cells responses to vaccines.

In conclusion, elucidating the mechanisms underlying poor immunological protection from vaccination due to transplantation and older age should result in more targeted interventions to improve vaccine responses [31]. The evaluation of T cell responses to influenza vaccination may be a more appropriate measure of protection in older or immunosuppressed adults. Indeed T cell responses were more predictive of protection than antibody titers in older adults [39]. Despite the limitations, our study provides the basis for future multi-center follow-up to test the effect of latent CMV on capacity for viral neutralization and to evaluate the expansion of TE + EM in future mechanistic studies of vaccine responsiveness in solid organ transplant recipients.

## Supplementary Material

1

## Figures and Tables

**Table 1:** Patient characteristics stratified by CMV status.

	All (n = 16)	CMV^+^ (n = 11)	CMV^−^ (n = 5)
Age*	70.5 ± 10	64 ± 13	73.3 ± 1
Years post transplantation*	3.5 ± 7.5	4 ± 7	4.4 ± 13
Sex	Male (100 %)	Male (100 %)	Male (100 %)
Vaccination in each of previous 3 years	8 (50 %)	6 (55 %)	2 (40 %)
Immunosuppressive therapy at vaccination	16 (100 %)	11 (100 %)	5 (100 %)
Anti-metabolite	15 (94 %)	10 (91 %)	5 (100 %)
Calcineurin Inhibitor	14 (88 %)	9 (82 %)	5 (100 %)
Steroid	13 (81 %)	10 (91 %)	3 (60 %)
Other	2 (13 %)	2 (18 %)	0 (0 %)

* Median ± interquartile range.

**Table 2:** Influenza vaccination outcomes stratified by CMV status.

	All (n = 16)	CMV^+^ (n = 11)	CMV^−^ (n = 5)
Pre-vaccination IgG*	589.6 +/− 255.1	623.6 +/− 392.8	545.4 +/− 336.7
Δ IgG*	118.0+/−192.3	108.9 +/− 144.5	127.1 +/− 285
Pre-vaccination FRNT90 titer^+^	12.5 ± 12.5	20 ± 25	10 ± 5
ΔFRNT90 titer^+^	7.5 ± 22.5	10 ± 25	5 ± 10
Pre-vaccination FRNT90 titer ≥ 40	3 (19 %)	3 (27 %)	0 (0 %)
mFRNT90 titer > 40	7 (44 %)	6 (54 %)	1 (20 %)
Total number of samples evaluated	80	55	25

+ Median serum dilution ± interquartile range.

## Appendix A. Supplementary data

Supplementary data to this article can be found online at https://doi.org/10.1016/j.humimm.2026.111721.

## Abbreviations:

PBMC: Peripheral Blood Mononuclear Cells
T_E+EM_: Effector and Effector memory T cells
T_TEMRA_: T-cell Effector Memory Re-expressing RA
T_CM_: T Central memory
T_Naive_: Naïve T cells
CMV: Cytomegalovirus
KLRG1: Killer cell lectin-like receptor G1
FACS: Fluorescence-Activated Cell Sorting
FRNT90: Focus Reduction Neutralization Titer 90 test
mFRNT90: maximum Focus Reduction Neutralization Titer 90
ΔFRNT90: Difference Focus Reduction Neutralization Titer 90
KTR: Kidney Transplant Recipient
