# Supplementary material for: Pre-existing influenza antibodies, younger age, and increased CD4 TE+EM predict influenza vaccination responses in transplant recipients
Source: Hum Immunol. Author manuscript; Available in PMC 2026 Jun 2. (PMC13227456; doi:10.1016/j.humimm.2026.111721)
Supplement: 1 [file NIHMS2179598-supplement-1.docx]

**SUPPLEMENTAL TABLE 1**


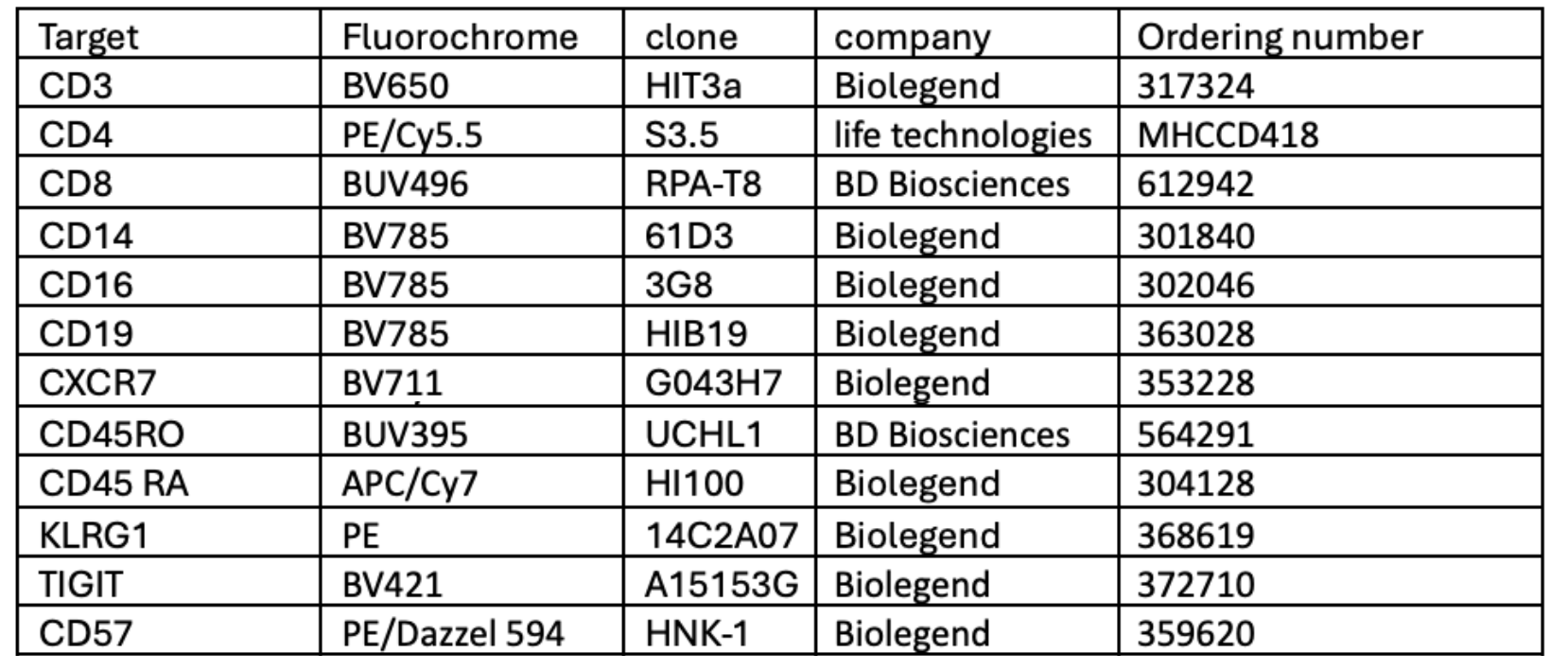


**Antibody table for FACS experiments**

In addition to the listed antibodies, cells were also stained with Zombie Aqua dye (Biolegend, San Diego, CA) to gate out dead cells. Compensation controls were prepared by using eBioscience Ultracomp eBeads (San Diego, CA) and stained following manufacturer protocol.

**SUPPLEMENTAL FIGURE 1**


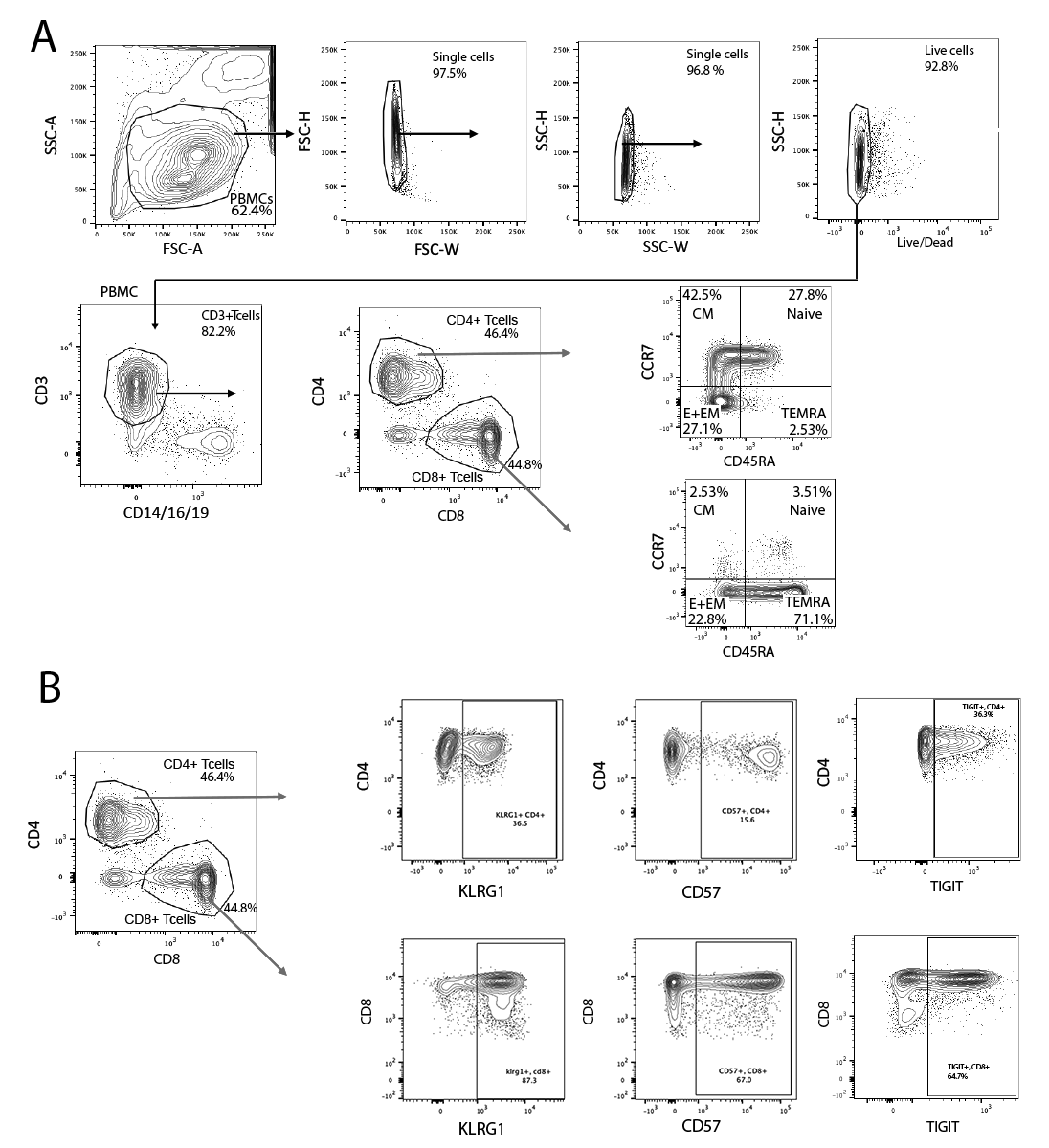


**Flow Cytometry gating strategy :** Example of FACS gating used for analysis of a participant’s PBMCs. (A) Lympohocyte gated PBMCs were subsequently gated on singlets and live cells. Live, singlet gated PBMCs were then gated on CD3+CD19- T cells then for CD4 and CD8. CD4+ and CD8+ T cells were then assessed for subsets including Naïve, T_CM_, T_E+EM_ and TEMRA utilizing CCR7 and CD45RA expression as shown. (B) Representative gating of CD4 and CD8 T cells assessed for CD57+, KLRG1+, and TIGIT+ populations. Relative percentage of cells within each gate is noted in all plots.

**SUPPLEMENTAL FIGURE 2**


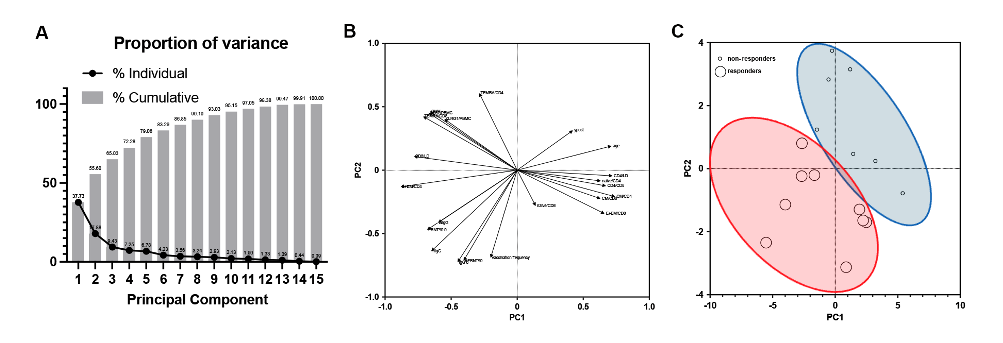


**Principal Component Analysis of all flow cytometry and demographic variables.**

(A) Bar graph depicting proportion variance with the individual and cumulative Eigenvalues are shown for each PC. PC1 and PC2 account for 38% and 8% of the variance, respectively. (B) Biplot of PC1 and PC2 illustrating variable loadings, where arrows represent key variables, with longer arrows indicating stronger correlation with PCs. (C) Score plot of PC1 and PC2 showing individual non-responder (blue small circle), and responder (red large circle) groups.

**SUPPLEMENTAL FIGURE 3**
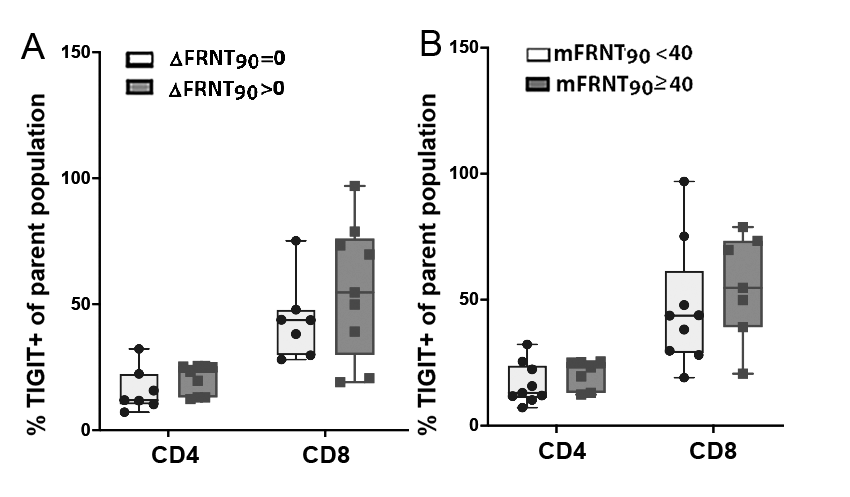
**Immune response and mFRNT90 values are not associated with the proportion of TIGIT+ CD4 or CD8 T cells.**

Boxplots show the distribution of (A) ΔFRNT90 and (B) mFRNT90 stratified by the percent of TIGIT positive cells. Boxplots show individual data points, with median and interquartile range.

p-values are based on the Mann-Whitney test *= p<0.05. n=16
